# The *Mycobacterium tuberculosis* Transposon Sequencing Database (MtbTnDB): A Large‐Scale Guide to Genetic Conditional Essentiality

**DOI:** 10.1111/mmi.15370

**Published:** 2025-06-17

**Authors:** Adrian Jinich, Anisha Zaveri, Michael A. DeJesus, Amanda Spencer, Ricardo Almada‐Monter, Emanuel Flores‐Bautista, Clare M. Smith, Christopher M. Sassetti, Jeremy M. Rock, Sabine Ehrt, Dirk Schnappinger, Thomas R. Ioerger, Kyu Y. Rhee

**Affiliations:** ^1^ Skaggs School of Pharmacy and Pharmaceutical Sciences UC San Diego La Jolla California USA; ^2^ Department of Chemistry and Biochemistry UC San Diego La Jolla California USA; ^3^ Department of Microbiology and Immunology Weill Cornell Medical College New York City New York USA; ^4^ Laboratory of Host‐Pathogen Biology The Rockefeller University New York City New York USA; ^5^ Division of Biology and Biological Engineering California Institute of Technology Pasadena California USA; ^6^ Department of Molecular Genetics and Microbiology Duke University Durham North Carolina USA; ^7^ Department of Microbiology and Physiological Systems University of Massachusetts Medical School Worcester Massachusetts USA; ^8^ Department of Computer Science and Engineering Texas A&M University College Station Texas USA; ^9^ Division of Infectious Diseases, Weill Department of Medicine Weill Cornell Medical College New York City New York USA

**Keywords:** database, functional genetics, microbiology, transposon sequencing, tuberculosis

## Abstract

Characterizing genetic essentiality across various conditions is fundamental for understanding gene function. Transposon sequencing (TnSeq) is a powerful technique to generate genome‐wide essentiality profiles in bacteria and has been extensively applied to *Mycobacterium tuberculosis* (*Mtb*). Dozens of TnSeq screens have yielded valuable insights into the biology of *Mtb* in vitro, inside macrophages, and in model host organisms. Despite their value, these *Mtb* TnSeq profiles have not been standardized or collated into a single, easily searchable database. This results in significant challenges when attempting to query and compare these resources, limiting our ability to obtain a comprehensive and consistent understanding of genetic conditional essentiality in *Mtb*. We address this problem by building a central repository of publicly available *Mtb* TnSeq screens, the *Mtb* transposon sequencing database (MtbTnDB). The MtbTnDB is a living resource that encompasses to date ≈150 standardized TnSeq screens, enabling open access to data, visualizations, and functional predictions through an interactive web app (www.mtbtndb.app). We conduct several statistical analyses on the complete database, such as demonstrating that (i) genes in the same genomic neighborhood have similar TnSeq profiles, and (ii) clusters of genes with similar TnSeq profiles are enriched for genes from similar functional categories. We further analyze the performance of machine learning models trained on TnSeq profiles to predict the functional annotation of orphan genes in *Mtb*. By facilitating the comparison of TnSeq screens across conditions, the MtbTnDB will accelerate the exploration of conditional genetic essentiality, provide insights into the functional organization of *Mtb* genes, and help predict gene function in this important human pathogen.

## Graphical abstract

*Mtb* transposon sequencing database is a unified resource for exploring conditional gene essentiality in *Mycobacterium tuberculosis* . By standardizing over 150 transposon sequencing screens, it enables rapid comparison of genetic dependencies across diverse experimental conditions and provides open access to data, visualizations, and functional predictions through an interactive web app.

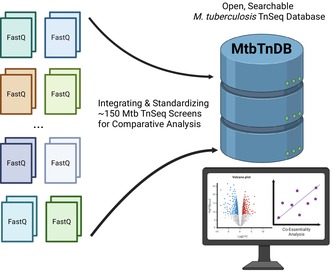

## 1 Introduction

Assessing the essentiality profile of a gene across different conditions is often a useful first step toward determining its function. When determined for all genes in an organism, similar patterns of essentiality across a set of conditions can indicate a shared or common function, as in the case of enzymes in a metabolic pathway (Griffin et al. 2011). Evidence of such shared or related functions can be further informed by constructing gene–gene interaction networks consisting of genes that are essential on background mutant strains that harbor single or multiple gene deletions (DeJesus, Nambi, et al. 2017; van Opijnen et al. 2015). In the context of pathogenic bacteria, genes that are essential for virulence in clinically relevant conditions represent potential high‐priority targets for drug development (Jacobs et al. 2003; Salama et al. 2004).

For many bacterial organisms, including the human pathogen *Mycobacterium tuberculosis* (*Mtb*), one of the main approaches to investigate the effect of gene knockouts on fitness at genome scale is to generate pooled transposon insertion mutant libraries and measure the relative growth or survival via transposon sequencing (TnSeq) (van Opijnen et al. 2015; Chao et al. 2016; Cain et al. 2020). In TnSeq, a saturated transposon insertion library is generated and cultured in a defined experimental condition. Amplification and sequencing of the output and control libraries yield, for each genomic region, a ratio of experiment‐to‐control library insertion counts. These insertion ratios are analyzed statistically in order to categorize genes as conditionally essential or nonessential (DeJesus et al. 2015; DeJesus, Nambi, et al. 2017; Barquist et al. 2016).

Two decades ago, Rubin et al. (1999) adapted transposon mutagenesis, coupled to microarray‐based genome‐wide fitness measurements, to *Mtb*. This method was subsequently modified using next‐generation sequencing to allow single base‐pair mapping of insertions (Griffin et al. 2011; Zhang et al. 2012). Since then, TnSeq has been used to identify *Mtb* conditionally essential genes using in vitro cultures, in vivo mouse models as well as macrophage infection assays, and in a wide variety of conditions, such as different carbon sources, stress conditions, and a diverse array of *Mtb* clinical and mutant strains (Table 1) (Griffin et al. 2011; DeJesus, Nambi, et al. 2017; Zhang et al. 2012, 2013; DeJesus and Ioerger 2013; Kieser et al. 2015; Mendum et al. 2015; Nambi et al. 2015; Korte et al. 2016; Xu et al. 2017; Mishra et al. 2017; Carey et al. 2018; Rittershaus et al. 2018; Bellerose et al. 2019; Minato et al. 2019; Smith et al. 2022; Meade et al. 2023). Unfortunately, these valuable genetic datasets remain scattered in supplementary tables throughout the literature, making rapid analysis of the conditional essentiality profiles of genes of interest time‐consuming. Additionally, a lack of analytical standardization makes comparison difficult even when studies are identified, as it is often not clear how to properly compare essentiality calls that are made with different statistical approaches.

**TABLE 1:** The space of experimental conditions covered by the *Mtb* TnSeq database.

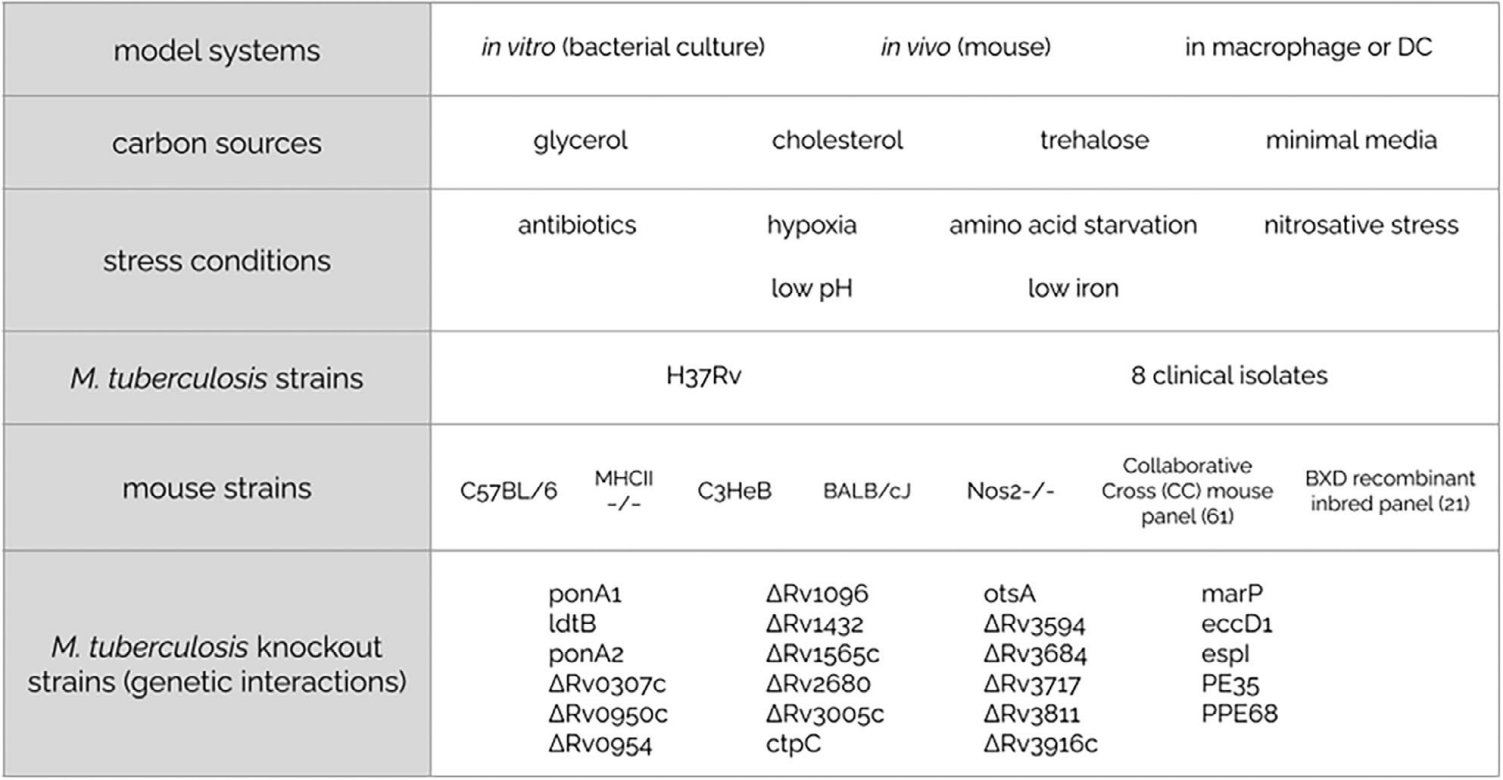

*Note:* This table includes the diverse conditions which Tnseq screening was performed under, including model systems, carbon sources, stress conditions, *Mtb* strains, mouse strains, and *Mtb* knockout strains used in studies to investigate genetic interactions.

**FIGURE 1:**
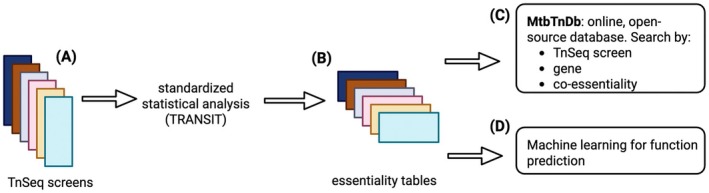
Schematic diagram of compilation and standardization of *M. tuberculosis* TnSeq screens. (A) Raw sequencing reads were collected from publicly available TnSeq screens in the literature and the FLUTE database. (B) Using a standardized statistical processing framework with TRANSIT (DeJesus et al. 2015), each set of raw reads was converted into an essentiality table, with genes in the *Mtb* genome assigned log2 fold‐change values (relative to specified control conditions) and *p*‐values corrected for multiple hypothesis testing. (C) The set of TnSeq data can be queried in the MtbTnDB online portal, either by screen or by gene of interest. (D) The database is amenable to both supervised and unsupervised machine learning approaches to generate hypotheses about gene function.

To address this gap, we compiled a wide array of publicly available *M. tuberculosis* TnSeq datasets into a single standardized, open‐access database, which we call the *Mtb* transposon sequencing database (MtbTnDB) (Figure 1). The MtbTnDB contains 158 unique screens from 21 different publications and from the Functionalizing Lists of Unknown TB Entities (FLUTE) database (orca2.tamu.edu/U19), covering a range of experimental and physiological conditions (Table 1). To help facilitate access to the database for the TB research community, we developed an online tool that allows querying the database by either TnSeq screen or gene of interest and provides informative analyses and visualizations of the compiled data. We illustrate the potential value of the MtbTnDB for TB research through example applications, such as easily identifying orphan (i.e., unannotated) genes that are conditionally essential in a given TnSeq screen. Finally, we apply unsupervised and supervised machine learning techniques to extract insights into the function of genes from their TnSeq profiles.

## 2 Materials and Methods

### 2.1 Standardization of TnSeq Datasets

To analytically standardize and process available TnSeq raw sequencing data, we selected TRANSIT, a Python‐based TnSeq analysis tool, and Tn‐Seq preprocessor (TPP), a software tool for processing raw reads (DeJesus et al. 2015). We obtained either original raw sequencing data or read‐counts in a format suitable for analysis, such as Wiggle Track Format (.wig), for 146 out of the 158 TnSeq screens in the database. For conditions where raw reads were available, we first used TPP, a preprocessing tool included with TRANSIT, to map them all to the genome sequence of the standard H37Rv reference strain and obtain .wig formatted files.

Those datasets for which we could not obtain raw sequencing data (12 screens) were not included for analysis but are referenced in the MtbTnDB website to allow users to look at their results. As an additional quality control step, we only included screens for which the transposon insertion density was higher than 25%. Raw reads were mapped with TPP to the H37Rv genome using default settings. We note that for screens that did not use H37Rv, genes that are deleted in the experimental strain but not in the control strain—or vice versa—may cause false positive conditional essentiality calls. The resulting .wig files produced by TPP were analyzed using the resampling in TRANSIT, which compares two experimental conditions to find significantly different read counts in a gene. We also included pseudocounts in the resampling pipeline, using the default of PC = 1 in TRANSIT. Whenever possible, a condition (experiment) was compared to the reference dataset (control) specified in a TnSeq screen's source publication. For publications that did not specify a reference or control condition, a highly saturated in vitro dataset (DeJesus, Gerrick, et al. 2017) was used. Resampling was run using default settings, using TTR normalization and 10,000 permutations. *p*‐values were automatically adjusted for multiple hypothesis testing by TRANSIT using the Benjamini–Hochberg procedure.

We labeled genes as conditionally essential in a given TnSeq screen if the following two conditions were satisfied: *p*‐val (adjusted) ≤ 0.05 and *abs*(log2FC) ≥ 1, where $\log2\mathrm{FC}=\mathrm{lo}g_{2}\left(\frac{\text{experiment}+\text{pseudocounts}}{\text{control}+\text{pseudocounts}}\right)$, that is, the logarithm base 2 of the ratio of insertion counts (adding the fixed so‐called pseudocounts parameter), and *abs* is the absolute value. We note that the use of the absolute value in the threshold condition results in some selected genes having positive log2FC values, meaning they are detected as significantly *less* essential than expected by chance in that particular condition, with their loss providing a fitness *advantage*.

To inspect the TnSeq screens for potential batch effects, each TnSeq screen was represented by an *N*‐dimensional vector of log2FC (where *N* is the number of genes in the *Mtb* genome) and we performed dimensionality reduction of these vectors using the uniform manifold approximation and projection (UMAP) algorithm.

### 2.2 MtbTnDB Website and Open‐Source Code

The MtbTnDB online portal was built using Dash (version 1.14.0), a tool for web‐based analytical apps in Python. All source code related to analyses mentioned in this work is available at https://github.com/ajinich/mtb_tn_db/ while all source code used to build the online portal is available at https://github.com/ajinich/mtb_tn_db_demo.

### 2.3 Classification of Genes Into Annotation Categories

We classified genes in the H37Rv genome into a set of five categories ranging from least to most well characterized using annotation scores from the UniProt Knowledgebase (UniProt Consortium 2019), which evaluate available experimental evidence for each gene at the protein level.

### 2.4 Clustering of Genes With UMAP

To perform the unsupervised learning analysis of the standardized TnSeq profiles using UMAP, we first removed all genes (2285) that have zero conditional essentiality calls across all 147 standardized screens. We then used the standardized binary TnSeq profiles for the remaining number of genes input into UMAP (with two components), as implemented in Python. Having obtained the UMAP components, we performed K‐means clustering in the two‐dimensional projection, selecting the optimal number of clusters that maximizes the Silhouette Coefficient (Rousseeuw 1987). We used Fisher's (hypergeometric) enrichment test to look for potential enrichments of COG or Tuberculist annotation functional categories within each cluster. Finally, to further evaluate the statistical significance of the observed enrichments within clusters, we generated randomized versions of the UMAP dataset by fixing the UMAP coordinates and cluster identities, shuffling the gene identifiers and annotation information associated with each point in the UMAP projection, and repeating the statistical enrichment test.

### 2.5 Prediction of Gene Function

To fit a supervised machine learning model to the standardized TnSeq profiles, we used the log2FC values from the standardized matrix as inputs. Genes containing missing values in some screens were dropped (*n* = 84). We used Tuberculist annotation functional categories as classification labels and dropped genes belonging to the “unknown” (*n* = 14) or “conserved hypothetical” (*n* = 1020) categories. The resulting matrix containing 2937 genes was used for prediction. Additionally, we used SMOTE to balance the labels in the classification task (Lemaître et al. 2017).

## 3 Results

### 3.1 Compiling and Standardizing *M. tuberculosis* TnSeq Datasets

We set out to build a database that compiles all publicly available *M. tuberculosis* TnSeq datasets in a standardized, open‐source format (Figure 1). We assembled data for *Mtb* TnSeq screens encompassing a total of 158 comparisons between experimental and control conditions from two main sources. A first set of 143 comparisons was compiled from 21 publications (Griffin et al. 2011; DeJesus, Nambi, et al. 2017; Zhang et al. 2012, 2013; DeJesus and Ioerger 2013; Kieser et al. 2015; Mendum et al. 2015; Nambi et al. 2015; Korte et al. 2016; Xu et al. 2017; Mishra et al. 2017; Carey et al. 2018; Rittershaus et al. 2018; Bellerose et al. 2019; Minato et al. 2019; Smith et al. 2022; Meade et al. 2023; Sassetti et al. 2003; Sassetti and Rubin 2003; Rengarajan et al. 2005; Joshi et al. 2006). The remaining 15 were obtained from the FLUTE database (orca2.tamu.edu/U19).

The set of unique TnSeq screens in the MtbTnDB cover a wide range of different experimental conditions (Table 1). Of the 158 screens in the database, 47 are in vitro bacterial culture experiments, covering many different culture media (carbon sources and limiting nutrients) and stress conditions (antibiotics, low oxygen tensions, acidic pH, nitrosative stress, iron limitation, and amino acid starvation). A second set of 109 screens is in vivo experiments using mouse infection models. Two recent publications (Smith et al. 2022; Meade et al. 2023) are the source of the large majority (82 out of 109) of these in vivo screens. An additional two TnSeq screens use cell culture (macrophage and dendritic cell) infection models. Although the large majority of the experiments were performed using the standard laboratory *Mtb* strain (H37Rv), one publication (Carey et al. 2018) performed comparative screens on eight different clinical isolates. A number of the TnSeq comparisons (34 unique TnSeq screens) were performed on *Mtb* strains harboring a single gene knockout, yielding information on genetic interaction networks underlying the observed conditional essentiality profiles. Finally, for completion, we also included data for five screens from the literature that were performed using microarray‐based transposon site hybridization (Sassetti et al. 2003; Sassetti and Rubin 2003; Rengarajan et al. 2005; Joshi et al. 2006).

### 3.2 Standardized Statistical Analysis of TnSeq Screens Using TRANSIT

In order to facilitate comparative analyses across different TnSeq screens, we set out to reprocess the raw data for as many screens as possible using a single standardized statistical method based on TRANSIT, a Python‐based TnSeq analysis tool (see Section 2 for details). Although TRANSIT accommodates a variety of statistical approaches for making essentiality calls, we utilized the resampling method to compare every experimental condition to an appropriate reference or control condition. Briefly, resampling compares read counts between two different conditions utilizing a permutation test to identify significant differences in the mean read counts (DeJesus et al. 2015). Resulting hits are labeled as *conditionally* essential genes: genes that are essential in one background condition (experiment) but not another (reference or control). By comparing against a suitable reference or control condition, we sought to reduce potential biases that can exist due to differences in mutant libraries. Whenever possible, the reference condition was chosen to be the one described in the original TnSeq screen. When no suitable reference or control condition was specified or included in the screen, we utilized a fully saturated in vitro dataset (DeJesus, Gerrick, et al. 2017) as the control condition.

In order to analyze the effect of our standardized processing of the underlying TnSeq sequencing data, we compared the number of conditionally essential genes detected in each screen (using fixed threshold values for the corrected *p*‐value ≤ 0.05, and a log(2)‐fold change ≥ 1) against those reported in the corresponding original, individual datasets (Table S1). Across all screens analyzed, we find that the standardized data and the original analyses differ in around 2% of the conditional essentiality calls. Approximately half (or 1% of all conditional essentiality calls) of these are cases where the standardized dataset suggests that a gene is conditionally essential in a particular screen, whereas the original analysis did not, and the other half of the cases correspond to the opposite situation. A few individual screens have a relatively high number of non‐consensus essentiality calls between the standardized and original datasets. In particular, one TnSeq screen in an in vivo mouse model (Zhang et al. 2013) has 403 genes that were called essential in the original datasets, but not in the standardized analysis. One possible reason behind this large inconsistency is the different statistical methods used to analyze the sequencing data. We note that both the original and the standardized data sets are included in the MtbTnDB (see section on online database).

We next inspected the standardized TnSeq screens in search of potential batch effects. Dimensionality reduction of data vectors representing each TnSeq screen (Section 2) revealed two distinct clusters, one of which was largely dominated by a set of screens of a library of H37Rv conducted in a strain of C57Bl/6 mice by Meade et al. (Figure S1, clusters labeled Smith_2022 and Meade_2023). These screens were included as part of a study examining the impact of host loci on *Mtb* gene fitness across a panel of immunodivergent mice wherein C57Bl/6 mice were included as a *Mtb*‐resistant haplotype. Interestingly, the C57Bl/6 strain was also included in other TnSeq screens of H37Rv in our database. Comparing the TnSeq profiles from C57B1/6 mice in this screen to those reported by a separate study utilizing the same library and mice purchased from the same vendor examining different mouse panel strains revealed a nearly 10‐fold difference in bacterial load taken at the same time point following infection (Smith et al. 2022; Meade et al. 2023). The segregated clustering of all TnSeq profiles across all mouse strains from this study, including C57Bl/6, thus suggested an experimental source of batch effect.

### 3.3 The Statistics of Conditional Essentiality Across the MtbTnDB

**FIGURE 2:**
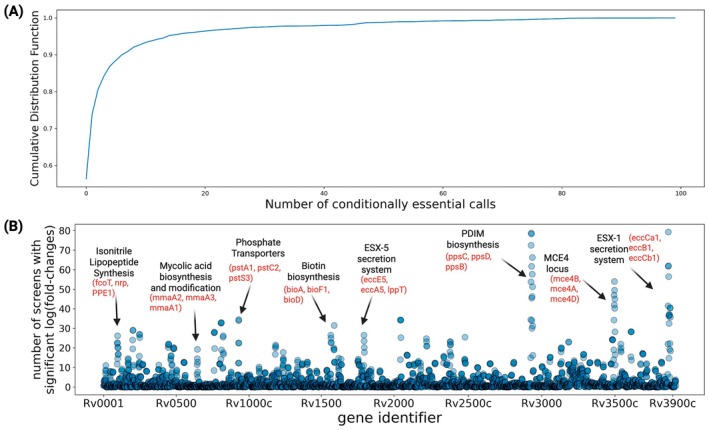
Distribution of conditional essentiality calls in the MtbTnDB across all genes. (A) Cumulative distribution of the number of conditionally essential calls across all genes in the *Mtb* genomes (B) Number of conditional essentiality calls versus position in the genome across the MtbTnDB for all H37Rv *Mtb* genes. The darker color of some areas indicates genes points overlapping each other.

Figure 2A shows the cumulative distribution of conditional essentiality calls across all genes in the *Mtb* genome. It shows the percentage of *Mtb* genes that are essential in at most N conditions. We find that 2285 genes are not labeled as conditionally essential in any of our standardized screens. We note that a significant subset of these (359) are genes that are essential or have a significant growth defect in the fully saturated in vitro screen (DeJesus, Gerrick, et al. 2017) that we used as a control (reference) condition for a large number of comparisons. Thus, despite being essential for growth in standard in vitro culture conditions, these genes are not detected as conditionally essential in any screen in the MtbTnDB: that is, there is no significant difference in their degree of essentiality in standard in vitro culture in comparison to the experimental conditions covered in the database. The rest of the genes with zero conditionally essential calls likely represent a mixture of putatively dispensable genes and (i) genes with essential roles in experimental conditions that are outside the coverage of our database, (ii) false negatives, that is, genes that do not meet the threshold of statistical significance due to experimental factors like low coverage.

At the tail of the distribution, we find approximately 296 genes that are conditionally essential in 10 or more comparative TnSeq screens. Among these (Figure 2B) we find a strong enrichment for phthiocerol synthesis polyketide synthase type I genes (*ppsA‐E*), with all members of these PDIM biosynthetic pathway genes having at least 76 conditional essentiality counts. One possible explanation for this is the fact that the loss of PDIM biosynthesis genes in vitro confers a fitness advantage to *Mtb* (Domenech and Reed 2009). The fully saturated in vitro dataset (34 unique TnSeq screens) that was used as the control condition for a large number of comparisons contains PDIM biosynthesis genes, but some of these genes could have been lost in several of the experimental conditions, leading to few or no detectable transposon insertion counts and resulting in false‐positive conditionally essential calls. Other gene loci with a high number of conditionally essential calls are the ESX‐1 secretion system (e.g., eccCa1, eccB1, eccCb1) the Mce‐4 locus (e.g., mce4B, mce4A, mce4D), and the isonitrile lipopeptide biosynthetic operon (e.g., fcoT, nrp, PPE1). Figure 2B shows a Manhattan plot style visualization highlighting the most prominent loci according to conditional essentiality counts.

### 3.4 MtbTnDB, an Online Database of Standardized *Mtb* TnSeq Screens

**FIGURE 3:**
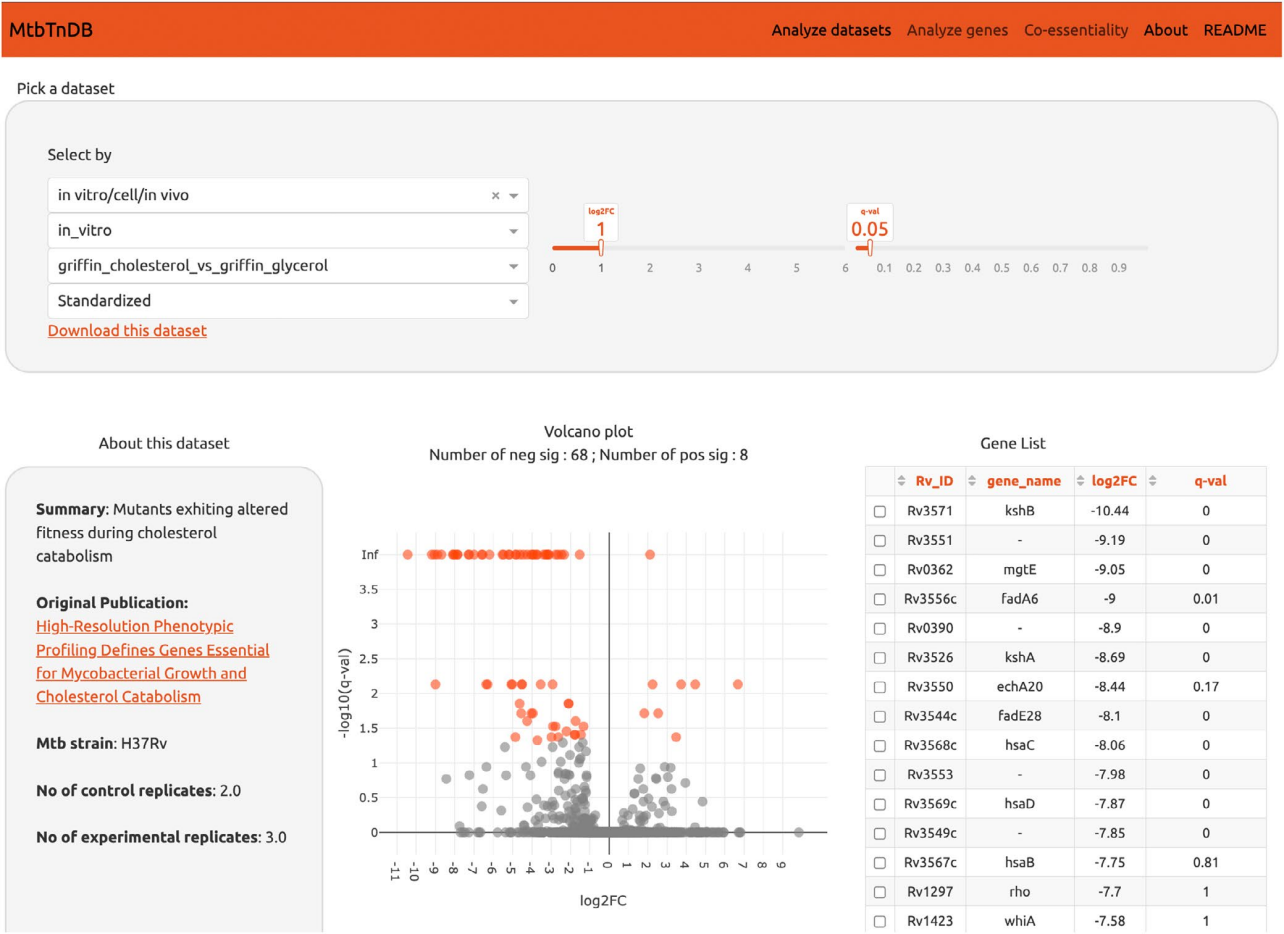
An online explorer of TnSeq datasets. A screenshot of the “Analyze datasets” modality is shown. Users can select which TnSeq screen to explore, whether to display either the standardized or the original publication's dataset, as well as significance thresholds for log2 fold‐changes and *q*‐values. A brief summary of the TnSeq screen is shown in the left panel, alongside a link to the original publication and the number of replicates for both control and experimental conditions. An interactive volcano plot highlights conditionally essential genes, which can also be accessed and selected through the table on the right.

To allow efficient querying of the standardized data, we developed an MtbTnDB web interface (www.mtbtndb.app). The interface allows three methods of querying the database—by screen (Figure 3), by gene, and in co‐essentiality mode. Each screen is a pairwise comparison between an experimental and control condition. The “Analyze datasets” tab provides an information‐rich and interactive overview of the selected comparison. An “About this dataset” section includes a description of the screen, links to the original publication, and the number of replicates used for both the experimental and control conditions. A volcano plot and accompanying table allow interrogation into differentially represented mutants. Sliders for significant log2FC and *q*val cut‐offs allow interactive coloring of data points on the volcano plot. Mutants of interest can also be highlighted on the volcano plot by selection in the accompanying table. We also provide two plots depicting broader analyses of the data. First, a bar plot depicts the enrichment of COG functional categories in overrepresented/underrepresented mutants. Second, a bubble plot allows identification of the subset of genes that are both conditionally essential in this analysis and the least annotated subset, providing useful pointers for follow‐up experiments. An example case using this type of plot is provided in the next section.

The second functionality provided in the web interface is to query genes of interest and list the set of TnSeq screens in which mutants for this gene are differentially represented. In addition to log2FC and *q*‐values, we also provide track views, which are visual depictions of the magnitude and location of insertions within the selected gene.

The third tab in the MtbTnDB web interface serves as a dedicated co‐essentiality analysis feature, which identifies significant correlations between the TnSeq profiles of gene pairs—specifically, the log2‐fold changes—across all experimental screens within the database. This functionality allows researchers to select a gene of interest and engage with an interactive visualization that shows significant correlations with other genes, suggesting potential functional interactions and dependencies. This visual and interactive interface displays both first‐and second‐degree co‐essential relationships, providing a comprehensive map of potential genetic interactions.

The data used in MtbTnDB are provided in the “About” tab, and users can also choose to download individual screens from the “Analyze datasets” tab. We also note the modularity of the database and its ability to accommodate additional datasets in the future, potentially including conditional essentiality screens using CRISPRi.

### 3.5 Example Usage: Selecting Experimental Conditions to Study Unannotated Genes

A large fraction of genes in the *Mtb* genome remains unannotated, and the MtbTnDB could help annotate such “orphan” genes by guiding the design of experimental conditions in which to assay their function. This is motivated by the notion that experimental conditions in which a mutant strain displays a growth or survival defect are more likely to reveal the orphan gene's physiological role. For instance, if an enzyme of unknown function participates in cholesterol catabolism, the probability of discovering its function will be highest if the mutant strain's metabolic profile is obtained when grown with cholesterol as its primary carbon source.

**FIGURE 4:**
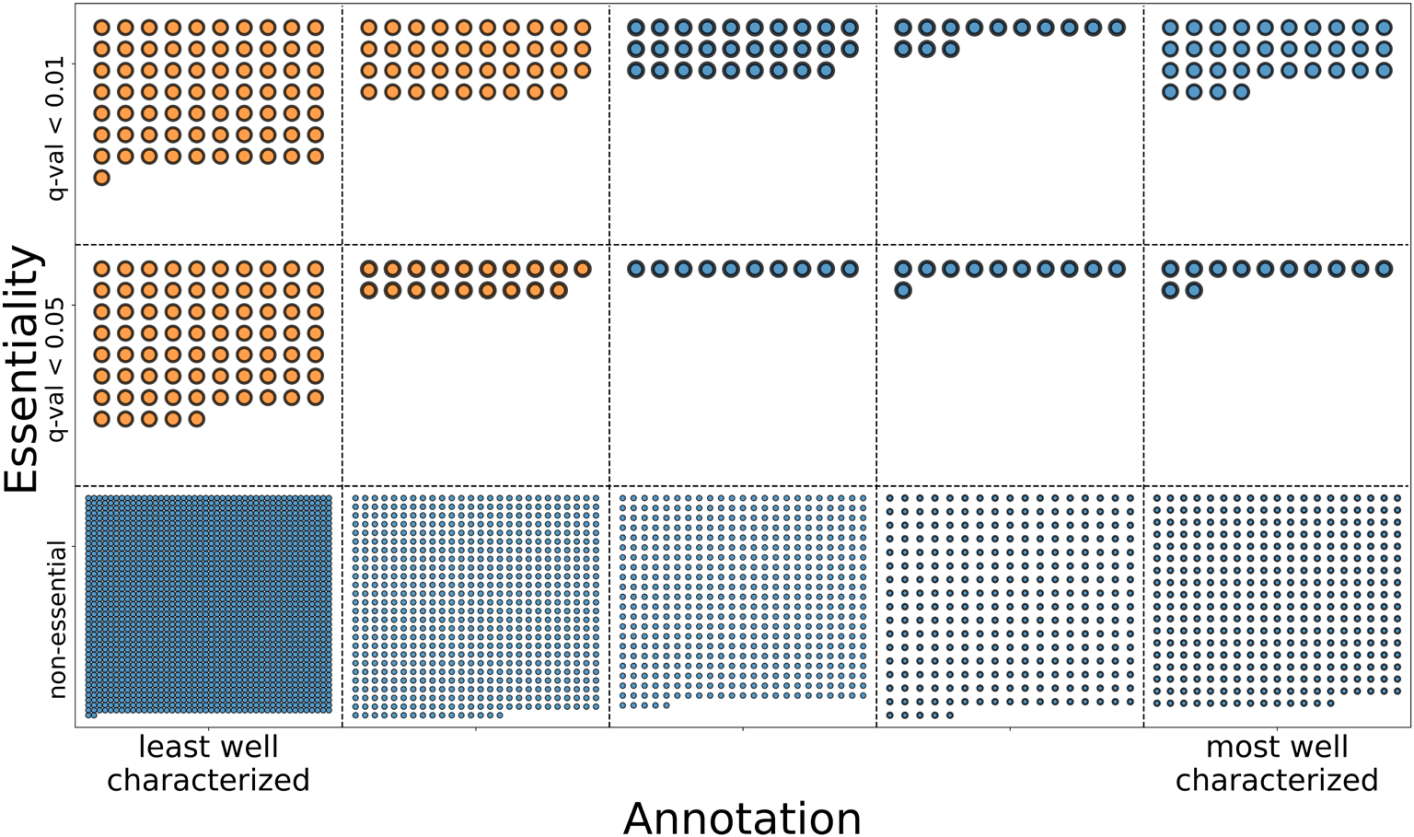
Mapping out conditionally essential genes of unknown function. Data shown correspond to a single TnSeq screen in the MtbTnDB: An H37Rv in vivo (mouse) screen (DeJesus, Nambi, et al. 2017). Genes are represented as circles, and are binned along the *x*‐axis into five distinct categories according to their annotation level and along the *y*‐axis according to their conditional essentiality (the exact position within each bin/category is not meaningful). The least well characterized genes (i.e., orphans) that are conditionally essential in this particular TnSeq screen are shown in orange (the gene identifiers for five example genes are shown). These five highlighted genes represent random samples of the orphan genes found to be conditionally essential within this TnSeq screen condition.

To help select experimental conditions in which to study orphan genes, we used experimental annotation scores from Uniprot, consisting of five categories ranging from least to most well characterized (see Section 2), to classify genes. For every TnSeq screen in the MtbTnDB, we then obtain the unknown essentials: the set of genes that are both conditionally essential (in that experimental screen) and fall in the category of least well characterized genes, so‐called “orphans” some of which are identified with their RvIDs in the figure (Figure 4, top‐left corner). The number of unknown conditional essentials varies across each TnSeq screen in the MtbTnDB, with 29 screens having more than 30 conditionally essential genes with the lowest annotation score. In particular, 10 screens have more than 50 conditionally essential genes that fall in the most poorly annotated category, underscoring the fact that the function of many potential drug targets remains to be discovered. This also illustrates how the MtbTnDB can be used to systematically select experimental conditions that result in measurable growth phenotypes for orphan genes of interest for further study.

### 3.6 Predicting Functional Categories With the MtbTnDB

**FIGURE 5:**
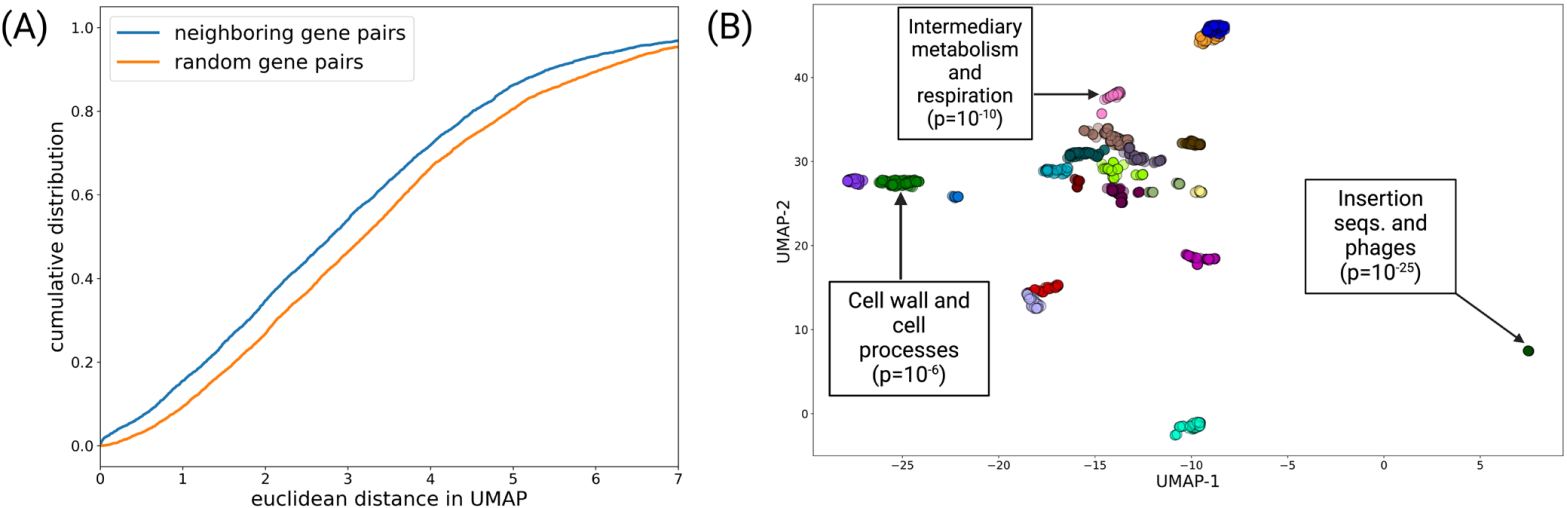
Genes with similar TnSeq profiles tend to have similar functions. Neighboring genes in the genome have more similar TnSeq profiles, and clusters of genes with similar TnSeq profiles are enriched for annotated genes belonging to the same functional categories. (A) Distribution of Euclidean distances (in UMAP projection) for pairs of random genes (orange) and neighboring genes (i.e., less than 3 genes away from each other in the genome). The two distributions are significantly different (*p* = 10^−17^, Kolmogorov–Smirnov test). (B) A UMAP of TnSeq profiles. Genes with at least one conditional essentiality call in the MtbTnDB were clustered using k‐means according to their UMAP coordinates and color‐coded. Three clusters enriched for gene functional categories are shown.

We asked whether similar TnSeq conditional essentiality profiles are indicative of similar biological function, where a conditional essentiality profile is defined as the set of (conditional) essentiality calls across all standardized TnSeq screens; that is, a 147‐dimensional vector for each gene in the *Mtb* genome. To explore this, we applied UMAP, a nonlinear dimensional reduction tool (McInnes et al. 2018), to the standardized TnSeq data. We hypothesized that, since genes belonging to the same operons are functionally related, neighboring genes in the genome are likely to have more similar TnSeq conditional essentiality profiles than random pairs of genes on average (Figure 5A). To test this, we compared the distribution of Euclidean distances in the UMAP projection between (i) pairs of genes that are in the same genomic neighborhood (i.e., apart from each other in the genome by a fixed cutoff genomic distance) and (ii) pairs of genes chosen at random from the full *Mtb* genome. We indeed find that neighboring genes tend to have more similar TnSeq profiles than expected by chance (*p* = 10^−17^, Kolmogorov–Smirnov test).

Next, using k‐means clustering, we grouped genes by the similarity of their TnSeq profiles as captured by their coordinates in the UMAP projection. An example UMAP projection with 21 different clusters is shown in (Figure 5B). Using the COG (Tatusov et al. 2000) and Tuberculist (Lew et al. 2011) *Mtb* genome annotations, we find that several clusters are significantly enriched for a number of different functional categories, including, “intermediary metabolism and respiration,” “insertion seqs. and phages,” and “cell wall and cell processes.” Importantly, our observation of clusters enriched for functional categories is statistically significant (*p* < 0.05, permutation test), and on average, zero categories were enriched when annotations were shuffled and reanalyzed for overlap with the UMAP clusters (Figure S2). We note that the tight clustering of “insertion sequences and phages” is due to the identical nature of their sequences, which results in artifactually identical effect sizes (log2FC) in the TnSeq analysis pipeline. However, taken together, these analyses serve as evidence that the TnSeq profiles capture functional relationships between genes, and encode information about the underlying operonic structure of the *Mtb* genome.

**FIGURE 6:**
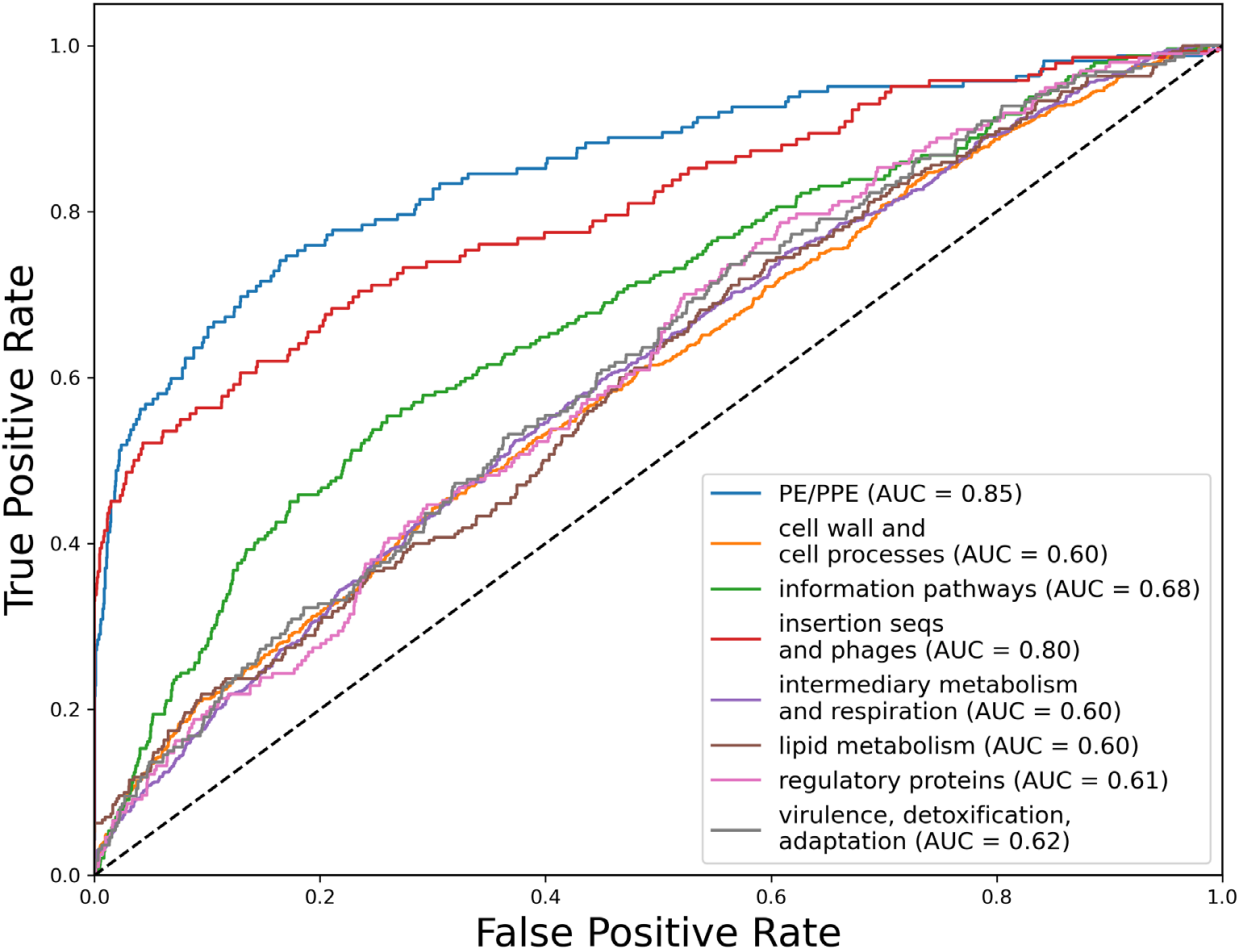
Mapping TnSeq essentiality profiles to gene functional categories with machine learning classifier. The receiver operating characteristic (ROC) curve for each functional class is shown. Multilabel predictions were generated using XGBoost (Chen and Guestrin 2016). The number of genes in each functional category are: PE/PPE (162); cell wall and cell processes (771); information pathways (242); insertion sequences and phages (142); intermediary metabolism and respiration (933); lipid metabolism (270); regulatory proteins (197); and virulence, detoxification, adaptation (220).

Building on the unsupervised analysis described above, we reasoned that since genes with similar physiological functions have similar essentiality profiles, the information encoded in the *Mtb* TnSeq Matrix could be used to help computationally annotate orphan genes. Toward this end, we trained a multilabel classification model which takes as input a gene's log2FC values from the 147 screens in the database and predicts whether a gene belongs to each of eight different functional categories, as defined in the Tuberculist genome annotation database (Lew et al. 2011). An XGBoost classifier achieved an area under the receiver operating characteristic (ROC) curve (AU‐ROC) of between 0.69 and 0.86 (fivefold cross‐validation) for three Tuberculist categories: “PE/PPE,” “information pathways,” and ‘insertion sequences and phages’ (Figure 6). We note that (i) our prediction is completely blind to the coding sequence, and that (ii) as a comparative baseline, the accuracy of an unbiased random classifier would have an AU‐ROC value of 0.5. Thus, while improvements in predictive accuracy can be achieved by incorporating other sources of data, TnSeq essentiality profiles can potentially help guide the functional annotation of unknown genes in *Mtb*. In addition, the predictive power of machine learning models trained on TnSeq data will likely improve as the database is populated with more conditional essentiality screens.

## 4 Discussion

To facilitate access to and the ability to gain insight from the vast amount of information stored in *M. tuberculosis* genetic essentiality screens, we compiled a large set of publicly available TnSeq screens into a single, standardized, and open‐access database, the MtbTnDB. While other TnSeq databases exist (Luo et al. 2021), these contain significantly fewer Mtb‐specific screens and do not address the issue of standardization of raw sequencing data. We envision periodically updating the MtbTnDB by adding publicly available TnSeq screens as they become available in the literature: researchers wishing to contribute their screens to the database would simply need to provide the sequence files along with a basic description of the screening conditions.

Although we aimed to maximize the degree of standardization, we note that there are upstream experimental factors that introduce variability into the screens, placing inherent limits in our ability to standardize the data. For instance, the transposon libraries differ in saturation (the number of unique insertion sites with one or more mutants present in the library), or in protocol details, such as whether barcoding was utilized, or the specific sequencing technology used to obtain the reads. These differences may introduce artifacts, such as PCR overamplification, or simply reduce the amount of data that is available (in the case of libraries with low coverage), which ultimately will affect the statistical power of downstream analyses.

One limitation of the current set of TnSeq screens included in the MtbTnDB is a bias toward a subspace of experimental conditions. Future efforts should therefore be directed toward covering a wider and more diverse region of experimental condition space. Toward this end, a recent TnSeq screen assaying iron uptake in *M. tuberculosis* (Zhang et al. 2020), as well as recent technical developments that couple TnSeq with random DNA barcoding (RB‐TnSeq) allowing parallel screening across dozens of conditions (Price et al. 2018) are very encouraging.

It is worthwhile to point out that the information afforded by TnSeq experiments is quite different and complementary from that of RNAseq (transcriptomics). Transcriptomics identifies sets of genes whose expression changes in response to changes in conditions, which can be clustered into sets of co‐regulated genes (or regulons) and sometimes correlated with transcription factors (DeJesus, Nambi, et al. 2017; Rustad et al. 2014). There is a large amount of transcriptomic data (DNA microarrays and RNAseq) that has been collected for *Mtb* in different conditions over the years, including stress conditions like hypoxia and antibiotic exposure, in macrophages and mouse models, knockout strains, among others (Moretto et al. 2016). TnSeq identifies the essentiality of genes in a given condition. In the extreme, essential genes are those that are indispensable for growth in the condition and hence cannot tolerate transposon insertions. Additionally, there might be some genes that exhibit a quantitative reduction in transposon insertion counts, which is interpreted to indicate a fitness defect (growth impairment) caused by the disruption of the ORF (Sassetti et al. 2003). Hence, TnSeq reveals information about which genes play functional roles in pathways required for survival in the condition. While both technologies can be efficiently scaled up to profile genome‐wide cellular responses to changes in environmental conditions, the assays assess different information about genes (essentiality vs. expression), and the lists of hits (conditionally essential genes vs. differentially regulated genes) often exhibit little overlap (Jensen et al. 2017). A gene can be differentially expressed without being conditionally essential and, conversely, a gene can become conditionally essential without a significant change in expression in the new condition. Efforts to combine TnSeq and RNAseq to enhance function prediction are ongoing.

The prospect of combining high‐throughput phenotypic screening with machine learning to illuminate orphan gene function is promising. In this work, we tested how well the MtbTnDB could functionally classify unannotated *Mtb* genes using supervised machine learning models and found limited prediction accuracy. One possible explanation for this could be the quality of the functional annotations themselves, and future directions would include combining the MtbTnDB with complementary sources of data, such as gene expression datasets, in order to improve the prediction of orphan gene function. We envision that the essentiality patterns encoded in the collected and standardized TnSeq screens will help researchers unravel the higher‐order, physiological function of genes in the *Mtb* genome.

Our initial release of MtbTnDB includes a large collection of standardized TnSeq screens, though we acknowledge there are additional valuable TnSeq studies in the field, such as those examining early infection dynamics (Payros et al. 2021) and biofilm formation (Richards et al. 2019). Many of these studies use compatible methodological approaches and will be incorporated in future updates of the database. As a living resource, MtbTnDB is designed to grow alongside the field, and we actively encourage researchers to contribute their TnSeq datasets. This continuous expansion will help build an increasingly comprehensive picture of conditional essentiality across diverse physiological conditions in Mtb.

To ensure direct comparability with existing datasets, new studies contributed to MtbTnDB should adhere to key experimental and analytical considerations. First, raw sequencing data should be available in FASTQ format and processed using TRANSIT, following the same statistical pipeline detailed in this work. Insertion densities should exceed 25% genome coverage to ensure sufficient resolution for essentiality calls. Additionally, new datasets should include clearly defined control conditions, ideally aligning with those in existing screens to facilitate cross‐study comparisons. When control conditions are unspecified, a well‐characterized in vitro reference dataset can be used as a baseline. Metadata tracking is equally critical, including details on the strain background, experimental conditions, sequencing depth, and statistical parameters, all of which are systematically recorded in the MtbTnDB framework. Future updates will refine these standardization guidelines as additional datasets are integrated.

## Author Contributions

**Adrian Jinich:** conceptualization, investigation, writing – original draft, methodology, validation, visualization, writing – review and editing, software, formal analysis, project administration, data curation. **Anisha Zaveri:** conceptualization, methodology, software, writing – original draft, writing – review and editing, formal analysis, data curation. **Michael A. DeJesus:** conceptualization, investigation, methodology, validation, data curation, software, formal analysis. **Amanda Spencer:** software, writing – review and editing, data curation, formal analysis. **Ricardo Almada‐Monter:** software, visualization. **Emanuel Flores‐Bautista:** software. **Clare M. Smith:** data curation, writing – review and editing. **Christopher M. Sassetti:** data curation. **Jeremy M. Rock:** data curation, writing – review and editing. **Sabine Ehrt:** data curation, writing – review and editing. **Dirk Schnappinger:** data curation, writing – review and editing. **Thomas R. Ioerger:** data curation, writing – review and editing. **Kyu Y. Rhee:** conceptualization, investigation, funding acquisition, writing – original draft, resources, supervision, project administration.

## Conflicts of Interest

The authors declare no conflicts of interest.

## Supporting information


**Figure S1.** Unsupervised clustering of standardized TnSeq data by screen reveals a subset of screens with potential batch effects. Two‐dimensional projection of TnSeq screens was performed using the Uniform Manifold Approximation and Projection (UMAP) algorithm. Each point corresponds to a single TnSeq screen in the MtbTnDB, and screens are color‐coded according to the reference publication or data‐source.
**Figure S2.** Lack of enrichment of gene functional categories in randomized versions of gene essentiality data. To evaluate the statistical significance of the observed enrichments of COG and Tuberculist gene functional categories in UMAP projection of TnSeq essentiality profiles, we generated 200 randomized versions of the UMAP dataset. Specifically, the positions of genes in the 2‐dimensional UMAP projection were fixed, while the gene information (Rv‐ID, name, annotation) were randomly shuffled. The normalized histogram shows that in more than 95% of the shuffled datasets, no enrichment of functional categories was detected in any cluster.
**Table S1.** Consensus of conditional essentiality calls between standardized datasets and original published datasets.

## Data Availability

Software and source code are available GitHub: https://github.com/ajinich/mtb_tn_db.

## References

[mmi15370-bib-0001] Barquist, L. , M. Mayho , C. Cummins , et al. 2016. “The TraDIS Toolkit: Sequencing and Analysis for Dense Transposon Mutant Libraries.” Bioinformatics 32: 1109–1111. 26794317 10.1093/bioinformatics/btw022 PMC4896371

[mmi15370-bib-0002] Bellerose, M. M. , S.‐H. Baek , C.‐C. Huang , et al. 2019. “Common Variants in the Glycerol Kinase Gene Reduce Tuberculosis Drug Efficacy.” MBio 10: e00663‐19. 10.1128/mBio.00663-19. 31363023 PMC6667613

[mmi15370-bib-0003] Cain, A. K. , L. Barquist , A. L. Goodman , I. T. Paulsen , J. Parkhill , and T. van Opijnen . 2020. “A Decade of Advances in Transposon‐Insertion Sequencing.” Nature Reviews. Genetics 21: 526–540. 10.1038/s41576-020-0244-x PMC7291929 32533119

[mmi15370-bib-0004] Carey, A. F. , J. M. Rock , I. V. Krieger , et al. 2018. “TnSeq of *Mycobacterium tuberculosis* Clinical Isolates Reveals Strain‐Specific Antibiotic Liabilities.” PLoS Pathogens 14: e1006939. 29505613 10.1371/journal.ppat.1006939 PMC5854444

[mmi15370-bib-0005] Chao, M. C. , S. Abel , B. M. Davis , and M. K. Waldor . 2016. “The Design and Analysis of Transposon Insertion Sequencing Experiments.” Nature Reviews. Microbiology 14: 119–128. 26775926 10.1038/nrmicro.2015.7 PMC5099075

[mmi15370-bib-0006] Chen, T. , and C. Guestrin . 2016. “XGBoost: A Scalable Tree Boosting System.” KDD'16: Proceedings of the 22nd ACM SIGKDD International Conference on Knowledge Discovery and Data Mining. pp. 785–794. 10.1145/2939672.2939785.

[mmi15370-bib-0007] DeJesus, M. A. , C. Ambadipudi , R. Baker , C. Sassetti , and T. R. Ioerger . 2015. “TRANSIT—A Software Tool for Himar1 TnSeq Analysis.” PLoS Computational Biology 11: e1004401. 26447887 10.1371/journal.pcbi.1004401 PMC4598096

[mmi15370-bib-0008] DeJesus, M. A. , E. R. Gerrick , W. Xu , et al. 2017. “Comprehensive Essentiality Analysis of the *Mycobacterium tuberculosis* Genome via Saturating Transposon Mutagenesis.” MBio 8: e02133‐16. 10.1128/mBio.02133-16. 28096490 PMC5241402

[mmi15370-bib-0009] DeJesus, M. A. , and T. R. Ioerger . 2013. “A Hidden Markov Model for Identifying Essential and Growth‐Defect Regions in Bacterial Genomes From Transposon Insertion Sequencing Data.” BMC Bioinformatics 14: 303. 10.1186/1471-2105-14-303. 24103077 PMC3854130

[mmi15370-bib-0010] DeJesus, M. A. , S. Nambi , C. M. Smith , R. E. Baker , C. M. Sassetti , and T. R. Ioerger . 2017. “Statistical Analysis of Genetic Interactions in Tn‐Seq Data.” Nucleic Acids Research 45, no. 11: e93. 10.1093/nar/gkx128. 28334803 PMC5499643

[mmi15370-bib-0011] Domenech, P. , and M. B. Reed . 2009. “Rapid and Spontaneous Loss of Phthiocerol Dimycocerosate (PDIM) From *Mycobacterium tuberculosis* Grown In Vitro: Implications for Virulence Studies.” Microbiology 155: 3532–3543. 19661177 10.1099/mic.0.029199-0 PMC5154741

[mmi15370-bib-0012] Griffin, J. E. , J. D. Gawronski , M. A. Dejesus , T. R. Ioerger , B. J. Akerley , and C. M. Sassetti . 2011. “High‐Resolution Phenotypic Profiling Defines Genes Essential for Mycobacterial Growth and Cholesterol Catabolism.” PLoS Pathogens 7: e1002251. 21980284 10.1371/journal.ppat.1002251 PMC3182942

[mmi15370-bib-0013] Jacobs, M. A. , A. Alwood , I. Thaipisuttikul , et al. 2003. “Comprehensive Transposon Mutant Library of *Pseudomonas aeruginosa* .” Proceedings of the National Academy of Sciences of the United States of America 100, no. 24: 14339–14344. 10.1073/pnas.2036282100. 14617778 PMC283593

[mmi15370-bib-0014] Jensen, P. A. , Z. Zhu , and T. van Opijnen . 2017. “Antibiotics Disrupt Coordination Between Transcriptional and Phenotypic Stress Responses in Pathogenic Bacteria.” Cell Reports 20: 1705–1716. 28813680 10.1016/j.celrep.2017.07.062 PMC5584877

[mmi15370-bib-0015] Joshi, S. M. , A. K. Pandey , N. Capite , S. M. Fortune , E. J. Rubin , and C. M. Sassetti . 2006. “Characterization of Mycobacterial Virulence Genes Through Genetic Interaction Mapping.” Proceedings of the National Academy of Sciences of the United States of America 103: 11760–11765. 16868085 10.1073/pnas.0603179103 PMC1544243

[mmi15370-bib-0016] Kieser, K. J. , C. Baranowski , M. C. Chao , et al. 2015. “Peptidoglycan Synthesis in *Mycobacterium tuberculosis* Is Organized Into Networks With Varying Drug Susceptibility.” Proceedings of the National Academy of Sciences of the United States of America 112: 13087–13092. 26438867 10.1073/pnas.1514135112 PMC4620856

[mmi15370-bib-0017] Korte, J. , M. Alber , C. M. Trujillo , et al. 2016. “Trehalose‐6‐Phosphate‐Mediated Toxicity Determines Essentiality of OtsB2 in *Mycobacterium tuberculosis* In Vitro and in Mice.” PLoS Pathogens 12: e1006043. 27936238 10.1371/journal.ppat.1006043 PMC5148154

[mmi15370-bib-0018] Lemaître, G. , F. Nogueira , and C. K. Aridas . 2017. “Imbalanced‐Learn: A Python Toolbox to Tackle the Curse of Imbalanced Datasets in Machine Learning.” Journal of Machine Learning Research 18, no. 1: 559–563.

[mmi15370-bib-0019] Lew, J. M. , A. Kapopoulou , L. M. Jones , and S. T. Cole . 2011. “TubercuList – 10 Years After.” Tuberculosis 91: 1–7. 10.1016/j.tube.2010.09.008. 20980199

[mmi15370-bib-0020] Luo, H. , Y. Lin , T. Liu , et al. 2021. “DEG 15, an Update of the Database of Essential Genes That Includes Built‐In Analysis Tools.” Nucleic Acids Research 49: D677–D686. 33095861 10.1093/nar/gkaa917 PMC7779065

[mmi15370-bib-0021] McInnes, L. , J. Healy , N. Saul , and L. Großberger . 2018. “UMAP: Uniform Manifold Approximation and Projection.” Journal of Open Source Software 3: 861. 10.21105/joss.00861.

[mmi15370-bib-0022] Meade, R. K. , J. E. Long , A. Jinich , et al. 2023. “Genome‐Wide Screen Identifies Host Loci That Modulate *M. tuberculosis* Fitness in Immunodivergent Mice.” G3: Genes, Genomes, Genetics 13: jkad147. 10.1093/g3journal/jkad147. 37405387 PMC10468300

[mmi15370-bib-0023] Mendum, T. A. , H. Wu , A. M. Kierzek , and G. R. Stewart . 2015. “Lipid Metabolism and Type VII Secretion Systems Dominate the Genome Scale Virulence Profile of *Mycobacterium tuberculosis* in Human Dendritic Cells.” BMC Genomics 16: 372. 25956932 10.1186/s12864-015-1569-2 PMC4425887

[mmi15370-bib-0024] Minato, Y. , D. M. Gohl , J. M. Thiede , et al. 2019. “Genomewide Assessment of *Mycobacterium tuberculosis* Conditionally Essential Metabolic Pathways.” mSystems 4: e00070‐19. 10.1128/mSystems.00070-19. 31239393 PMC6593218

[mmi15370-bib-0025] Mishra, B. B. , R. R. Lovewell , A. J. Olive , et al. 2017. “Nitric Oxide Prevents a Pathogen‐Permissive Granulocytic Inflammation During Tuberculosis.” Nature Microbiology 2: 17072. 10.1038/nmicrobiol.2017.72 PMC5461879 28504669

[mmi15370-bib-0026] Moretto, M. , P. Sonego , N. Dierckxsens , et al. 2016. “COLOMBOS v3.0: Leveraging Gene Expression Compendia for Cross‐Species Analyses.” Nucleic Acids Research 44: D620–D623. 26586805 10.1093/nar/gkv1251 PMC4702885

[mmi15370-bib-0027] Nambi, S. , J. E. Long , B. B. Mishra , et al. 2015. “The Oxidative Stress Network of *Mycobacterium tuberculosis* Reveals Coordination Between Radical Detoxification Systems.” Cell Host & Microbe 17: 829–837. 26067605 10.1016/j.chom.2015.05.008 PMC4465913

[mmi15370-bib-0028] Payros, D. , H. Alonso , W. Malaga , et al. 2021. “Rv0180c Contributes to *Mycobacterium tuberculosis* Cell Shape and to Infectivity in Mice and Macrophages.” PLoS Pathogens 17, no. 11: e1010020. 10.1371/journal.ppat.1010020. 34724002 PMC8584747

[mmi15370-bib-0029] Price, M. N. , K. M. Wetmore , R. J. Waters , et al. 2018. “Mutant Phenotypes for Thousands of Bacterial Genes of Unknown Function.” Nature 557: 503–509. 29769716 10.1038/s41586-018-0124-0

[mmi15370-bib-0030] Rengarajan, J. , B. R. Bloom , and E. J. Rubin . 2005. “Genome‐Wide Requirements for *Mycobacterium tuberculosis* Adaptation and Survival in Macrophages.” Proceedings of the National Academy of Sciences of the United States of America 102: 8327–8332. 15928073 10.1073/pnas.0503272102 PMC1142121

[mmi15370-bib-0031] Richards, J. P. , W. Cai , N. A. Zill , W. Zhang , and A. K. Ojha . 2019. “Adaptation of *Mycobacterium tuberculosis* to Biofilm Growth Is Genetically Linked to Drug Tolerance.” Antimicrobial Agents and Chemotherapy 63: e01213‐19. 10.1128/AAC.01213-19. 31501144 PMC6811442

[mmi15370-bib-0032] Rittershaus, E. S. C. , S.‐H. Baek , I. V. Krieger , et al. 2018. “A Lysine Acetyltransferase Contributes to the Metabolic Adaptation to Hypoxia in *Mycobacterium tuberculosis* .” Cell Chemical Biology 25: 1495–1505.e3. 30318462 10.1016/j.chembiol.2018.09.009 PMC6309504

[mmi15370-bib-0033] Rousseeuw, P. J. 1987. “Silhouettes: A Graphical Aid to the Interpretation and Validation of Cluster Analysis.” Journal of Computational and Applied Mathematics 20: 53–65. 10.1016/0377-0427(87)90125-7.

[mmi15370-bib-0034] Rubin, E. J. , B. J. Akerley , V. N. Novik , D. J. Lampe , R. N. Husson , and J. J. Mekalanos . 1999. “In Vivo Transposition of Mariner‐Based Elements in Enteric Bacteria and Mycobacteria.” Proceedings of the National Academy of Sciences of the United States of America 96: 1645–1650. 9990078 10.1073/pnas.96.4.1645 PMC15546

[mmi15370-bib-0035] Rustad, T. R. , K. J. Minch , S. Ma , et al. 2014. “Mapping and Manipulating the *Mycobacterium tuberculosis* Transcriptome Using a Transcription Factor Overexpression‐Derived Regulatory Network.” Genome Biology 15: 502. 25380655 10.1186/s13059-014-0502-3 PMC4249609

[mmi15370-bib-0036] Salama, N. R. , B. Shepherd , and S. Falkow . 2004. “Global Transposon Mutagenesis and Essential Gene Analysis of *Helicobacter pylori* .” Journal of Bacteriology 186: 7926–7935. 15547264 10.1128/JB.186.23.7926-7935.2004 PMC529078

[mmi15370-bib-0037] Sassetti, C. M. , D. H. Boyd , and E. J. Rubin . 2003. “Genes Required for Mycobacterial Growth Defined by High Density Mutagenesis.” Molecular Microbiology 48: 77–84. 12657046 10.1046/j.1365-2958.2003.03425.x

[mmi15370-bib-0038] Sassetti, C. M. , and E. J. Rubin . 2003. “Genetic Requirements for Mycobacterial Survival During Infection.” Proceedings of the National Academy of Sciences of the United States of America 100: 12989–12994. 14569030 10.1073/pnas.2134250100 PMC240732

[mmi15370-bib-0039] Smith, C. M. , R. E. Baker , M. K. Proulx , et al. 2022. “Host‐Pathogen Genetic Interactions Underlie Tuberculosis Susceptibility in Genetically Diverse Mice.” eLife 11: e74419. 10.7554/eLife.74419. 35112666 PMC8846590

[mmi15370-bib-0040] Tatusov, R. L. , M. Y. Galperin , D. A. Natale , and E. V. Koonin . 2000. “The COG Database: A Tool for Genome‐Scale Analysis of Protein Functions and Evolution.” Nucleic Acids Research 28: 33–36. 10592175 10.1093/nar/28.1.33 PMC102395

[mmi15370-bib-0041] UniProt Consortium . 2019. “UniProt: A Worldwide Hub of Protein Knowledge.” Nucleic Acids Research 47, no. D1: D506–D515. 10.1093/nar/gky1049. 30395287 PMC6323992

[mmi15370-bib-0042] van Opijnen, T. , D. W. Lazinski , and A. Camilli . 2015. “Genome‐Wide Fitness and Genetic Interactions Determined by Tn‐Seq, a High‐Throughput Massively Parallel Sequencing Method for Microorganisms.” Current Protocols in Microbiology 36, no. 1: 1E.3.1–1E.3.24. 10.1002/9780471729259.mc01e03s36. PMC4696536 25641100

[mmi15370-bib-0043] Xu, W. , M. A. DeJesus , N. Rücker , et al. 2017. “Chemical Genetic Interaction Profiling Reveals Determinants of Intrinsic Antibiotic Resistance in *Mycobacterium tuberculosis* .” Antimicrobial Agents and Chemotherapy 61, no. 12: e01334‐17. 10.1128/AAC.01334-17. 28893793 PMC5700314

[mmi15370-bib-0044] Zhang, L. , R. C. Hendrickson , V. Meikle , E. J. Lefkowitz , T. R. Ioerger , and M. Niederweis . 2020. “Comprehensive Analysis of Iron Utilization by *Mycobacterium tuberculosis* .” PLoS Pathogens 16: e1008337. 32069330 10.1371/journal.ppat.1008337 PMC7058343

[mmi15370-bib-0045] Zhang, Y. J. , T. R. Ioerger , C. Huttenhower , et al. 2012. “Global Assessment of Genomic Regions Required for Growth in *Mycobacterium tuberculosis* .” PLoS Pathogens 8: e1002946. 23028335 10.1371/journal.ppat.1002946 PMC3460630

[mmi15370-bib-0046] Zhang, Y. J. , M. C. Reddy , T. R. Ioerger , et al. 2013. “Tryptophan Biosynthesis Protects Mycobacteria From CD4 T‐Cell‐Mediated Killing.” Cell 155: 1296–1308. 10.1016/j.cell.2013.10.045. 24315099 PMC3902092

